# Supporting the annotation of chronic obstructive pulmonary disease (COPD) phenotypes with text mining workflows

**DOI:** 10.1186/s13326-015-0004-6

**Published:** 2015-03-14

**Authors:** Xiao Fu, Riza Batista-Navarro, Rafal Rak, Sophia Ananiadou

**Affiliations:** National Centre for Text Mining, School of Computer Science, University of Manchester, Manchester Institute of Biotechnology, 131 Princess Street, Manchester, UK; Department of Computer Science, University of the Philippines Diliman, Quezon City, 1101 Philippines

**Keywords:** Corpus annotation, Phenotype curation, Automatic annotation workflows, Ontology linking, Corpora for clinical text mining, Chronic obstructive pulmonary disease

## Abstract

**Background:**

Chronic obstructive pulmonary disease (COPD) is a life-threatening lung disorder whose recent prevalence has led to an increasing burden on public healthcare. Phenotypic information in electronic clinical records is essential in providing suitable personalised treatment to patients with COPD. However, as phenotypes are often “hidden” within free text in clinical records, clinicians could benefit from text mining systems that facilitate their prompt recognition. This paper reports on a semi-automatic methodology for producing a corpus that can ultimately support the development of text mining tools that, in turn, will expedite the process of identifying groups of COPD patients.

**Methods:**

A corpus of 30 full-text papers was formed based on selection criteria informed by the expertise of COPD specialists. We developed an annotation scheme that is aimed at producing fine-grained, expressive and computable COPD annotations without burdening our curators with a highly complicated task. This was implemented in the Argo platform by means of a semi-automatic annotation workflow that integrates several text mining tools, including a graphical user interface for marking up documents.

**Results:**

When evaluated using gold standard (i.e., manually validated) annotations, the semi-automatic workflow was shown to obtain a micro-averaged F-score of 45.70% (with relaxed matching). Utilising the gold standard data to train new concept recognisers, we demonstrated that our corpus, although still a work in progress, can foster the development of significantly better performing COPD phenotype extractors.

**Conclusions:**

We describe in this work the means by which we aim to eventually support the process of COPD phenotype curation, i.e., by the application of various text mining tools integrated into an annotation workflow. Although the corpus being described is still under development, our results thus far are encouraging and show great potential in stimulating the development of further automatic COPD phenotype extractors.

**Electronic supplementary material:**

The online version of this article (doi:10.1186/s13326-015-0004-6) contains supplementary material, which is available to authorized users.

## Background

An umbrella term for a range of lung abnormalities, chronic obstructive pulmonary disease (COPD) pertains to medical conditions in which airflow from the lungs is repeatedly impeded. This life-threatening disease, known to be primarily caused by tobacco smoke, is not completely reversible and is incurable. COPD was ranked by the World Health Organization as the fifth leading cause of death worldwide in 2002, and is predicted to become the third by year 2030. Estimates have also shown that the mortality rate for COPD could escalate by at least 30% within the next decade if preventive measures are not implemented [1].

The disease and clinical manifestations of COPD are heterogeneous and widely vary from one patient to another. As such, its treatment needs to be highly personalised in order to ensure that the most suitable therapy is provided to a patient. COPD phenotyping allows for well-defined grouping of patients according to their prognostic and therapeutic characteristics, and thus informs the development and provision of personalised therapy [2].

The primary approach to recording phenotypic information is by means of electronic clinical records [3]. However, as clinicians at the point of care use free text in describing phenotypes, such information can easily become obscured and inaccessible [4]. In order to expedite the process of identifying a given patient’s COPD group, the phenotypic information locked away within these records needs to be automatically extracted and distilled for the clinicians’ perusal.

Capable of automatically distilling information expressed in natural language within documents, text mining can be applied on clinical records in order to efficiently extract COPD phenotypes of interest. However, the development of sophisticated text mining tools is reliant on the availability of gold standard annotated corpora, which serve as evaluation data as well as provide samples for training machine learning-based approaches.

This paper presents our ongoing efforts on the annotation of COPD phenotypes in a collection of scientific papers. In our previous publication [5] on which this work is built upon, we proposed to form a corpus of clinical records from the Multiparameter Intelligent Monitoring in Intensive Care II (MIMIC II) Clinical Database [6,7]. However, our UK-based expert collaborators (i.e., stakeholders who will incorporate our text mining technology into their systems in the near future) recently pointed out that there are substantial discrepancies between the hospital system in the US (on which MIMIC II is focussed) and that in the UK. After considering their advice, we decided to utilise scientific articles from various COPD-relevant journals, rather than build a corpus of clinical records which are highly US-specific. As previous work demonstrated techniques which successfully extracted information from unseen data even if the training/development data used was of a different document type [8], we believe that a gold standard corpus of full scientific articles should still allow for the development of phenotype extraction tools for clinical records. Nevertheless, our collaborators are still currently working on obtaining a subset of clinical records from their own hospital, which will also be annotated to become part of an augmented version of our corpus.

In embarking on this effort, we are building a resource that will support the development of text mining methods for the automatic extraction of COPD phenotypes from free text. We envisage that such methods will ultimately foster the development of applications which will enable point-of-care clinicians to more easily and confidently identify a given COPD patient’s group, potentially leading to the provision of the most appropriate personalised treatment. Furthermore, text mining methods can be employed in order to facilitate the linking of COPD phenotypes with genotypic information contained in published scientific literature.

In the remainder of this paper, we firstly provide a review of the state of the art (Related Work). We proceed to describing our methods for corpus development (Methods), including our strategy for document selection followed by our proposed annotation scheme. A discussion of our text mining-assisted annotation workflow is also provided. We then share the results and analysis of our evaluation (Results and Discussion). Lastly, we conclude the paper with a summary of our contributions and an overview of ongoing and future work.

### Related work

Various corpora have been constructed to support the development of clinical natural language processing (NLP) methods. Some contain annotations formed on the basis of document-level tags indicating the specific diseases that clinical reports pertain to. In the 2007 Computational Medicine Challenge data set [9], radiology reports were assigned codes from the ninth revision of the International Classification of Diseases-Clinical Modification (ICD-9-CM) terminology [10]. In similar corpora, chest X-ray reports were manually labelled with any of four pneumonia-related concepts [11] whilst any of 80 possible disease names were assigned to documents in another collection of clinical records [12] with the assistance of automatic tools MetaMap Transfer (MMTx) [13] for concept recognition and NegEx [14] for negation detection. Whilst suitable for evaluating information retrieval methods, such document-level annotations cannot sufficiently support the extraction of phenotypic concepts which are described in clinical records in largely variable ways, making it necessary for automated methods to perform analysis by looking at their actual mentions within text.

Several other clinical corpora were thus enriched with text-bound annotations, which serve as indicators of specific locations of phenotypic concept mentions within text. For instance, all mentions of signs or symptoms, medications and procedures relevant to inflammatory bowel disease were marked up in the corpus developed by South et al [15]. Specific mentions of diseases and signs or symptoms were similarly annotated under the ShARe scheme [16,17] and additionally linked to terms in the SNOMED Clinical Terms vocabulary [18]. Whilst the scheme developed by [19] had similar specifications, it is unique in terms of its employment of an automatic tool to accelerate the annotation process. One difficulty encountered by annotators following such scheme, however, is with manually mapping mentions of phenotypic concepts to vocabulary terms, owing to the high degree of variability in which these concepts are expressed in text. For instance, many signs or symptoms (e.g., *gradual progressive breathlessness*), cannot be fully mapped to any of the existing terms in vocabularies.

Alleviating this issue are schemes which were designed to enrich corpora with finer-grained text-bound annotations. The Clinical e-Science Framework (CLEF) annotation scheme [20] which defined several clinical concept types and relationships, required the decomposition of phrases into their constituent concepts which were then individually assigned concept type labels and linked using any of their defined relationships. Also based on a fine-grained annotation approach is the work by Mungall et al. [21] on the ontology-driven annotation of inter-species phenotypic information based on the EQ model [22]. Although their work was carried out with the help of the Phenote software [23] for storing, managing and visualising annotations, the entire curation process was done manually, i.e., without the support of any NLP tools. The effort we have undertaken, in contrast, can be considered as a step towards automating such EQ model-based fine-grained annotation of phenotypic information.

In this regard, our work is unique amongst annotation efforts within the clinical NLP community, but shares similarities with some phenotype curation pipelines employed in the domain of biological systematics. Curators of the Phenoscape project [24] manually link EQ-encoded phenotypes of fishes to the Zebrafish Model Organism Database using Phenex [25] which is a tool for managing character-by-taxon matrices, a formal approach used by evolutionary biologists. To accelerate this process, Phenex has been recently enhanced with NLP capabilities [26] upon the integration of a text analytic known as CharaParser [27]. Based on a combination of bootstrapping and syntactic parsing approaches [28], CharaParser can automatically annotate structured characteristics of organisms (i.e., phenotypes) in text, but currently does not have full support for linking concepts to ontologies [29]. Also facilitating the semi-automatic curation of systematics literature is GoldenGATE [30], a stand-alone application modelled after the GATE framework [31], which allows for the combination of various NLP tools into text processing pipelines. It is functionally similar to our Web-based annotation platform Argo [32] in terms of its support for NLP workflow management and manual validation of automatically generated annotations. However, the latter fosters interoperability to a higher degree by conforming to the industry-supported Unstructured Management Information Architecture [33] and allowing workflows to be invoked as Web services [34].

By producing our proposed fine-grained phenotype annotations which are linked to ontological concepts, we are representing them in a computable form thus making them suitable for computational applications such as inferencing and semantic search. The Phenomizer tool [35], for instance, has demonstrated the benefits of encoding phenotypic information in a computable format. Leveraging the Human Phenotype Ontology (HPO) [36] whose terms are linked to diseases in the Online Mendelian Inheritance in Man (OMIM) vocabulary [37], it supports clinicians in making diagnoses by semantically searching for the medical condition that best matches the HPO signs or symptoms given in a query. We envisage that such an application, when integrated with a repository of phenotypes and corresponding clinical recommendations, e.g., Phenotype Portal [38] and the Phenotype KnowledgeBase [39], can ultimately assist point-of-care clinicians in more confidently providing personalised treatment to patients. Our work on the annotation of COPD phenotypes aims to support the development of similar applications in the future.

## Methods

We describe in this section our strategies for collecting documents for the corpus and our proposed annotation scheme. We also elaborate on the technology behind our text mining-assisted annotation methodology.

### Document selection

**Figure 1:**
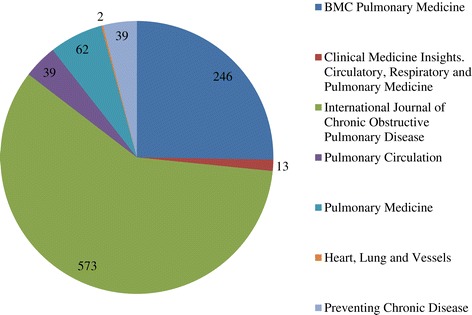
**Distribution of COPD-relevant articles over COPD-focussed journals.** A total of 974 full-text articles were retrieved from 10 journals in the PubMed OpenAccess subset.

In forming our corpus, we collected pertinent journal articles from the PubMed Central Open Access subset (PMC OA). As a preliminary step, we retrieved a list of journals which are most relevant to COPD by querying PMC OA using the keywords “chronic”, “obstructive”, “pulmonary”, “disease”, “respiratory” and “lung”. This resulted in ten journal titles whose archives were then searched for the keywords “chronic obstructive pulmonary disease” and “COPD”. A total of 974 full-text articles were retrieved in this manner. The journal titles and article distribution over them are shown in Figure 1.

Upon consideration of our constraints in terms of resources such as time and personnel, we decided to trim down the document set to 30 full articles. This was carried out by compiling a list of COPD phenotypes based on the combination of terms given by our domain experts and those automatically extracted by Termine [40] from the COPD guidelines published jointly by the American Thoracic Society and the European Respiratory Society in 2004 [41]. The resulting term list (provided as Additional file 1) contains 1,925 COPD phenotypes which were matched against the content of the initial set of 974 articles. In order to ensure that the documents in our corpus is representative of the widest possible range of COPD phenotypes, we ranked the documents according to decreasing number of their contained unique matches. We then selected the 30 top-ranked articles as the final document set for our corpus.

### A simple yet expressive annotation scheme

To capture and represent phenotypic information, we developed a typology of clinical concepts (Table 1) taking inspiration from the definition of COPD phenotypes previously proposed [2], i.e., “a single or combination of disease attributes that describe differences between individuals with COPD as they relate to clinically meaningful outcomes (symptoms, exacerbations, response to therapy, rate of disease progression, or death).” After reviewing the semantic representations used in previous clinical annotation efforts, we decided to adapt and harmonise concept types from the annotation schemes applied to the 2010 i2b2/VA Shared Task data set [42] and the PhenoCHF corpus [43]. In the former, concepts of interest were categorised into broad types of problem, treatment and test/measure. However, it was determined upon consultation with clinical experts that a finer-grained typology is necessary to better capture COPD phenotypes. For this, we looked into the semantic types used in the annotation of phenotypes for congestive heart failure in the PhenoCHF corpus, which are fine-grained yet generic enough to be applied to other medical conditions. We adapted some of those types and organised them under the upper-level types of the i2b2/VA scheme.

**Table 1:** **The proposed typology for capturing COPD phenotypes**

**Type**	**Description**	**Example(s)**
1) Problem	an overall category for any COPD indications of concern	*frequent exacerbator*
a) MedicalCondition*	any disease or medical condition; includes COPD comorbidities	*emphysema, pulmonary vascular disease, asthma, congestive heart failure*
b) RiskFactor*	a phenotype signifying a patient’s increased chances of having COPD	*increased levels of the c-reactive protein, alpha1 antitrypsin deficiency*
i) SignOrSymptom*	an observable irregularity manifested by a COPD patient	*chronic cough, shortness of breath, purulent sputum production*
ii) IndividualBehaviour*	a patient’s habits leading to susceptibility of having COPD	*smoking for 25 years*
iii) TestOrMeasureResult*	findings based on COPD-relevant examinations	*increased white blood cell counts, FEV1 45% predicted*
2) Treatment	any medication, therapy or program for treating COPD	*oxygen therapy, pulmonary rehabilitation, pursed lips breathing*
3) TestOrMeasure	an overall category for any COPD-relevant examinations or measures/parameters	*increased compliance of the lung, FEV1, FEV1/FVC ratio*
a) RadiologicalTest	any of the radiological tests for detecting COPD	*computed tomography scanning, high resolution computed tomography*
b) MicrobiologicalTest	an examination of a COPD- relevant specimen	*complete blood count*
c) PhysiologicalTest	a measurement of a COPD patient’s capacity to exercise	*6-min walking distance*

Types marked with an asterisk (*) were adapted from the PhenoCHF scheme.

Most phenotypes exemplified in Table 2 span full phrases, especially in the case of risk factors such as *increased compliance of the lung*, *chronic airways obstruction* and *increased levels of the c-reactive protein*. Some of the previously published schemes for annotating clinical text have proposed the encoding of phenotypes using highly structured, expressive representations. For the symptom expressed as *chronic airways obstruction*, for example, the CLEF annotation scheme [20] recommends its annotation to consist of a *has_location* relationship between *chronic obstruction* (a condition) and *airways* (locus). The EQ model for representing phenotypes [21], similarly, would decompose this phenotype into the following elements: *airways* as entity (E) and *chronic obstruction* as quality (Q). Whilst we recognise that such granular representations are ideal for the purposes of knowledge representation and automated knowledge inference, we feel that requiring them as part of the manual annotation of free-text documents significantly complicates the task for domain experts who may lack the necessary background in linguistics.

**Table 2:** **Examples of phenotypic information represented using our proposed annotation scheme**

**COPD Phenotypes**	**Automatically recognized underlying concepts**	**Automatically linked ontological concepts**
*chronic airways obstruction*	*chronic airways obstruction*	*chronic (PATO:0001863) respiratory airway (UBERON:0001005) obstructed (PATO:0000648)*
*parenchymal destruction*	*parenchymal destruction*	*parenchyma (UBERON:0000353) damaged (PATO:0001167)*
*decrease in rate of lung function*	*decrease in rate lung function*	*decreased rate (PATO:0000911) lung (UBERON:0002048) function (PATO:0000173)*
*chronic bronchitis*	N/A	*chronic bronchitis (DOID:6132)*
*myocardial infarction*	N/A	*myocardial infarction (DOID:5844)*
*enhanced response to inhaled corticosteroids*	*enhanced response to corticosteroids*	*enhanced (PATO:0001589) response to (PATO:0000077) corticosteroid (ChEBI:50858)*
*FEV1 45% predicted*	*FEV1*	*Forced Expiratory Volume 1 Test (NCIT:C38084)*
*alpha1 antitrypsin deficiency*	*alpha1 antitrypsin deficiency*	*alpha-1-antitrypsin (PR:000014678) decreased amount (PATO:0001997)*

We therefore propose an annotation methodology that strikes a balance between simplicity and granularity of annotations. On the one hand, our scheme renders the annotation task highly intuitive by asking for only simple text span selections, and not requiring the creation of relations nor the filling in of template slots. On the other hand, we also introduce granularity into the annotations by exploiting various semantic analytic tools, described in the next section, which automatically identify constituent ontological concepts. The contribution of applying automated concept identifiers is two-fold. Firstly, automatic concept identification as a pre-annotation step helps accelerate the manual annotation process by supplying visual cues to the annotators. For instance, the symptom expressed within text as *increased resistance of the small airways* becomes easier for an annotator to recognise, seeing that the elementary concepts *resistance* and *airways* have been pre-annotated. Secondly, as the constituent concepts will be linked to pertinent ontologies, the semantics of the expression signifying the symptom, which will be manually annotated as a simple text span, is nevertheless encoded in a fine-grained and computable manner. Shown in Table 2 are some examples of annotated phenotypes resulting from the application of our scheme.

### Text mining-assisted annotation with Argo

Our proposed methodology employs a number of text analytics to realise its aims of reducing the manual effort required from annotators and providing granular computable annotations of COPD phenotypes. After analysing several documents, we established that treatments are often composed of drug names (e.g., *Coumadin* in *Coumadin dosing*) whilst problems typically contain mentions of diseases/medical conditions (e.g., *myocardial infarction*), anatomical concepts (e.g., *airways* in *chronic airways obstruction*), proteins (e.g., *alpha1 antitrypsin* in *alpha1 antitrypsin deficiency*), qualities (e.g., *destruction* in *parenchymal destruction*) and tests (e.g., *FEV1* in *FEV1 45% predicted*). These observations, confirmed by COPD experts, guided us in selecting the automatic tools for recognising the above-mentioned types and for linking them to relevant ontologies.

**Figure 2:**
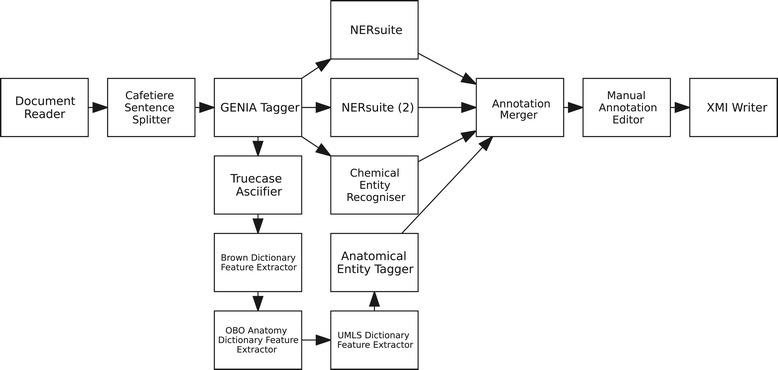
**Our semi-automatic annotation workflow in Argo.**

We used Argo [32], an interoperable Web-based text mining platform, to both integrate our elementary analytics into a processing workflow and to manage its execution. Argo’s rich library of processing components gives its users access to various text analytics ranging from data readers and writers to syntactic tools and concept recognisers. From these, we selected the components which are most suitable for our task’s requirements, and arranged them in a multi-branch automatic annotation workflow, depicted in Figure 2. The workflow begins with a Document Reader that reads the records from our corpus, followed by the Cafetiere Sentence Splitter which detects sentence boundaries. Resulting sentences are then segmented into tokens by the GENIA Tagger which also provides part-of-speech (POS) and chunk tags, and additionally recognises protein mentions [44].

**Figure 3:**
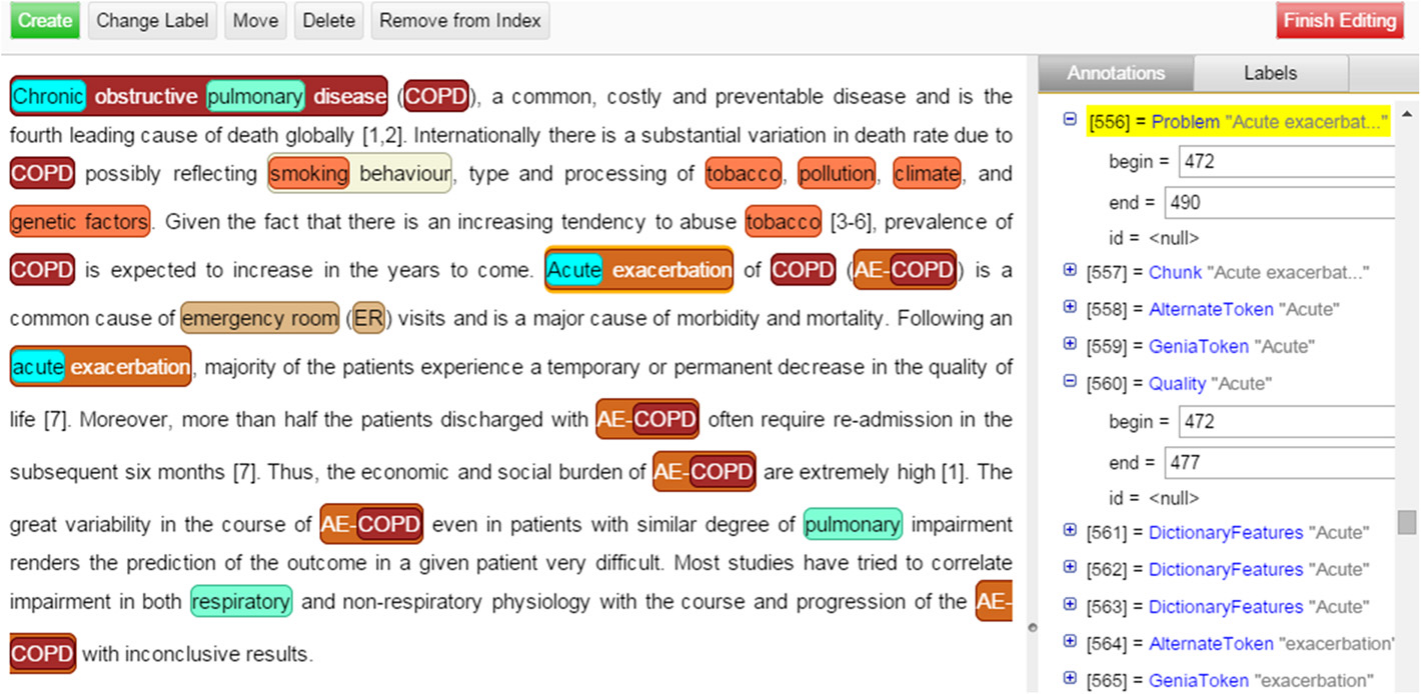
**The user interface for linking mentions to ontologies.**

After running the syntactic tools, the workflow splits into four branches. The first branch performs joint annotation of concepts pertaining to Problem, Treatment and TestOrMeasure by means of the NERsuite [45] component, a named entity recogniser (NER) based on an implementation of conditional random fields [46]. Supplied with a model trained on the 2010 i2b2/VA challenge training set [47], this NER is employed to provide domain experts with automatically generated cues which could aid them in marking up full phrases describing COPD phenotypes. Meanwhile, the NERsuite component in the second branch is configured to recognise disease mentions using a model trained on the NCBI Disease corpus [48]. The third branch performs drug name recognition using the Chemical Entity Recogniser, an adaptation of NERsuite employing chemistry-specific features and heuristics [49] which was parameterised with a model trained on the Drug-Drug Interaction (DDI) corpus [50]. Finally, by means of the Truecase Asciifier, Brown, OBO Anatomy and UMLS Dictionary Feature Extractors, the last branch extracts various features required by the Anatomical Entity Tagger which is capable of recognising anatomical concepts [51]. The Annotation Merger component collects annotations produced by the various concept recognisers whilst the Manual Annotation Editor allows human annotators to manually correct, add or remove automatically generated annotations via its rich graphical user interface (Figure 3).

Finally, the workflow’s last component, the XMI Writer, stores the annotated documents in the XML Metadata Interchange standard format, which allows us to reuse the output in other workflows if necessary. Eventually, the annotations can be made available in several other standard formats, such as RDF and BioC [52], which will be accomplished directly in Argo through its various serialisation components. We note that the automatic tool for recognising qualities is still under development, as are the components for linking mentions to concepts in ontologies. Nevertheless, we describe below our proposed strategy for ontological concept identification.

### Linking phenotypic mentions to ontologies

In order to identify the ontological concepts underlying COPD phenotypic information, the mentions automatically annotated by our concept recognisers will be normalised to entries in various ontologies, namely, the Phenotype and Trait Ontology (PATO) [53] for qualities, Human Disease Ontology (DO) [54] for medical conditions, Uber Anatomy Ontology (UBERON) [55] for anatomical entities, Chemical Entities of Biological Interest (ChEBI) [56] for drugs, Protein Ontology (PRO) [57] for proteins and the National Cancer Institute Thesaurus (NCIT) [58] for tests/measures.

The NCBO Annotator [59], formerly Open Biomedical Annotator, offers a solution to this problem by employing a Web service that automatically matches text against specific ontologies. It is, however, not sufficient for the requirements of our task as it is very limited in terms of variant-matching [60], obtaining only exact string matches against terms and synonyms contained in ontologies. As observed from the examples in Table 2, there is a large variation in the expressions comprising COPD phenotypes. Consequently, many of these expressions do not exist in ontologies in the same form. More suitable, therefore, is a sophisticated normalisation method that takes into consideration morphological variations (e.g., *alpha1 antitrypsin* vs. *alpha-1-antitrypsin*), inflections (e.g., *obstruction* vs. *obstructed*), syntactic variations (e.g., *decrease in rate* vs. *decreased rate*) and synonym sets (e.g., *deficiency* vs. *decreased amount* and *destruction* vs. *damage*).

**Figure 4:**
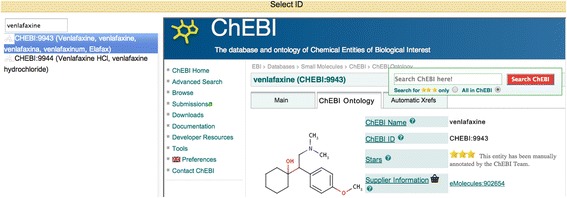
**The Manual Annotation Editor’s graphical user interface.** The article excerpt shown is annotated using our proposed scheme for finer-grained COPD phenotype annotations.

Argo’s library includes several automatic ontology-linking components employing approximate string matching algorithms [61]. Furthermore, the Manual Annotation Editor provides a user-friendly interface for manually supplying or correcting links to ontological concepts (Figure 4). Ongoing development work on improving this ontology-linking tool includes: (a) enhancement of the normalisation method by the incorporation of algorithms for measuring syntactic and semantic similarity, and (b) shifting from Argo’s currently existing ontology-specific linker components to a generic one that allows for linking mentions against any ontology (from a specified set). Once ready, the new component will be added to Argo’s library. Instances of the component will then be integrated into our semi-automatic workflow to facilitate the linking of annotated mentions to the respective ontologies.

## Results and discussion

After applying the Argo workflow described above on the 30 articles in our corpus, we asked one of our collaborating domain experts to manually validate the automatically generated annotations. In this section, we present the results of two types of evaluation. Firstly, the quality of the Argo-generated concept annotations was measured by comparing them against gold standard data, i.e., the annotations manually validated by the domain expert. Secondly, we carried out a preliminary evaluation of the gold standard annotations that we have obtained thus far by utilising them in the development of machine learning-based concept recognisers. It is worth noting that our gold standard data is currently limited to our expert’s annotations on only nine out of the 30 papers that she has examined thus far (equivalent to 1,701 sentences). Table 3 presents the number of unique concepts for each type, as manually annotated by our domain expert. One can see that the most prevalent types are Treatment, RiskFactor, MedicalCondition, TestOrMeasure, Drug and AnatomicalConcept (in order of decreasing frequency).

**Table 3:** **Number of unique concepts for each type, based on the nine manually annotated articles**

**Concept type**	**Number of unique concepts**
Treatment	430
RiskFactor	415
MedicalCondition	371
TestOrMeasure	282
Drug	192
AnatomicalConcept	96
Quality	59
Protein	40
**Total**	**1,885**

Table 4 depicts the evaluation of Argo’s automatically generated annotations against the gold standard, presented by concept type. We note that only the five most frequently occurring concept types (which are common between the manually validated annotations we have at hand and the automatically generated annotations) were included in the evaluation. Two different modes of matching were applied: exact matching, which considers a system annotation as correct only if it has the same concept type label and exactly the same boundaries as a gold standard annotation; and relaxed matching, which counts even a partially overlapping system annotation as correct as long the non-overlapping tokens consist of only articles and modifiers (i.e., they have only “DT”, “JJ” or “RB” as POS tags). We note that for a given phenotypic expression, not only the full string is being evaluated, but also each of its subsumed concepts. It can be observed from Table 4 that in general, the semi-automatic workflow obtains unsatisfactory performance using exact matching. After performing some error analysis, we observed that majority of discrepancies were brought about by the incorrect inclusion or exclusion of articles or modifiers in noun phrases, e.g., *phosphodiesterase inhibitor* (for *a nonselective phosphodiesterase inhibitor*), *an acute exacerbation* (for *acute exacerbation*). Thus we next employed relaxed matching, which revealed that the semi-automatic workflow obtains moderate performance over all evaluated concept types (except for TestOrMeasure).

**Table 4:** **Evaluation of annotations automatically generated by the text mining-assisted workflow against gold standard data**

	**Exact matching**			**Relaxed matching**		
	**Precision**	**Recall**	**F-score**	**Precision**	**Recall**	**F-score**
AnatomicalConcept	0.1923	0.7527	0.3063	0.2814	0.9038	0.4292
Drug	0.5861	0.2744	0.3738	0.7921	0.6463	0.7118
MedicalCondition	0.0290	0.2842	0.2868	0.3697	0.6313	0.4663
TestOrMeasure	0.1425	0.0680	0.0920	0.1914	0.1039	0.1347
Treatment	0.3080	0.1494	0.2012	0.4688	0.4015	0.4325
Micro-average	0.2670	0.2283	0.2462	0.4050	0.5243	0.4570
Macro-average	0.3037	0.3057	0.3047	0.4207	0.5374	0.4719

Results are reported for only nine full-text papers.

It is obviously more desirable for a semi-automatic workflow to approximate the gold standard annotations (i.e., to produce exact matches rather than partial ones). Nevertheless, Argo’s automatically generated annotations proved to be helpful in a number of cases. For example, the automatic workflow was able to correctly annotate partially correct annotations such as *sputum* (for *sputum smear*), *pulmonary* (for *pulmonary TB*) and *COPD-staging* (for *COPD*) served as visual cues to the annotator. Based on her experience in annotating our corpus, she feels that having pre-supplied annotations, albeit incomplete or incorrect, is preferable over not having any annotations at all. We are, however, aware of the potential bias that having pre-supplied annotations may bring about, i.e., failure to annotate concepts completely missed by automatic annotation due to reliance on visual cues. To avoid this scenario, the annotator has been asked to read all of the sentences thoroughly and to keep in mind that the cues are not to be relied on. She has adhered to this guideline throughout her annotations.

Applying the gold standard annotations to an information extraction task, we employed NERsuite, an implementation of the conditional random fields (CRFs) algorithm, to develop a new set of concept recognisers. Samples were represented using features which are by default extracted by NERsuite, including character, token, lemma and part-of-speech tag *n-*grams (within a distance of 2 from the token under consideration), chunk tags, as well as a comprehensive set of orthographic features (e.g., presence of uppercase or lowercase letters, digits, special characters). The resulting models were then evaluated in two ways. Firstly, for each concept type, models were trained and subsequently evaluated in a 10-fold cross-validation manner, whose results are presented in Table 5 alongside those obtained by the Argo components. In generating the folds, the articles were split at the paragraph level, giving a total of 381 shorter documents. Secondly, to facilitate evaluation on unseen data, each of the automatically and manually annotated subset of nine papers was subdivided into training (75% or 286 paragraphs) and held-out data (25% or 95 paragraphs). Models trained on the former were then evaluated using annotations contained in the latter. Table 6 presents the evaluation results under this setting.

**Table 5:** **Results of 10-fold cross validation of concept recognisers, using exact matching**

	**Concept recognisers currently in Argo**			**Concept recognisers trained on our corpus**		
	**Precision**	**Recall**	**F-score**	**Precision**	**Recall**	**F-score**
AnatomicalConcept	0.2361	0.6617	0.3428	0.7602	0.4990	0.5912
Drug	0.7318	0.2161	0.3283	0.8576	0.4499	0.5873
MedicalCondition	0.3986	0.2436	0.3010	0.8510	0.4590	0.5932
TestOrMeasure	0.0766	0.0182	0.0289	0.6850	0.3190	0.4332
Treatment	0.4330	0.1021	0.1635	0.8276	0.3458	0.4829
Micro-average	0.3305	0.1776	0.2310	0.7929	0.3970	0.5291
Macro-average	0.3752	0.2483	0.2988	0.7963	0.4145	0.5452

Performance is compared with that of the components utilised in the text mining-assisted workflow.

**Table 6:** **Results of evaluation using a fixed split over 381 paragraphs (training set: 75% or 286 paragraphs; held-out set: 25% or 95 paragraphs), using exact matching**

	**Concept recognisers currently in Argo**			**Concept recognisers trained on our corpus**		
	**Precision**	**Recall**	**F-score**	**Precision**	**Recall**	**F-score**
AnatomicalConcept	0.2602	0.6145	0.3656	0.8000	0.4314	0.5605
Drug	0.6885	0.1900	0.2979	0.7966	0.4196	0.5497
MedicalCondition	0.4494	0.2492	0.3206	0.8673	0.3899	0.5380
TestOrMeasure	0.0250	0.0041	0.0070	0.6719	0.2966	0.4115
Treatment	0.4111	0.0847	0.1404	0.8400	0.2903	0.4315
Micro-average	0.3735	0.1614	0.2254	0.8034	0.3552	0.4926
Macro-average	0.3669	0.2285	0.2816	0.7952	0.3656	0.5009

We show that by using our gold standard annotations as training data, we were able to develop concept recognisers whose performance is drastically better than those we employed in our semi-automatic workflow. This significant improvement ranged from 24.84 (for AnatomicalConcept) to 40.43 (for TestOrMeasure) percentage points according to 10-fold cross validation, and from 19.49 (for AnatomicalConcept) to 40.45 (for TestOrMeasure) according to the fixed split evaluation. This implies that our corpus can stimulate the development of more suitable automatic COPD phenotype extractors. We expect that as more gold standard annotations become available to us (i.e., as our domain expert completes the validation of more documents in our corpus), the better equipped we will be in boosting the performance of our automatic COPD concept recognisers.

## Conclusions

In this paper, we elucidate our proposed text mining-assisted methodology for the gold-standard annotation of COPD phenotypes in a corpus of full-text scientific articles. We demonstrate with the proposed scheme that the annotation task can be kept simple for curators whilst producing expressive and computable annotations. By constructing a semi-automatic annotation workflow in Argo, we seamlessly integrate and take advantage of several automatic NLP tools for the task. Furthermore, we are providing the domain experts with a highly intuitive interface for creating and manipulating annotations. The comparison of annotations automatically generated by the workflow against manually validated ones (i.e., gold standard) reveals an F-score of 45.70% using relaxed matching. New concept recognisers trained on these gold standard annotations demonstrate dramatically better performance (i.e., with a 20- to 30-percentage point margin in terms of F-scores) over the off-the-shelf components used in the Argo workflow.

Manual expert validation of the text mining-generated annotations on the remaining 21 papers in the corpus is still ongoing. In the meantime, we are enhancing our ontology concept linkers, which, once ready, will be applied on the gold standard concepts to enrich our corpus with computable annotations. Our expert collaborators are also working hard on obtaining a subset of clinical records from their hospital, which will then be used to augment our corpus. With the resulting resource, which will be made publicly available upon completion, we aim to support the development and evaluation of text mining systems that can ultimately be applied to evidence-based healthcare and clinical decision support systems.

## Additional file

Additional file 1:
**List of COPD phenotypes used to retrieve articles from the PubMed OpenAccess subset.**
